# LncRNA SNHG1 enhances cartilage regeneration by modulating chondrogenic differentiation and angiogenesis potentials of JBMMSCs via mitochondrial function regulation

**DOI:** 10.1186/s13287-024-03793-2

**Published:** 2024-06-18

**Authors:** Hua Liu, Huina Liu, Qiubo Yang, Zhipeng Fan

**Affiliations:** 1 Beijing Stomatological Hospital, School of Stomatology, Capital Medical University, Beijing, China; 2 Beijing Key Laboratory of Tooth Regeneration and Function Reconstruction, Beijing Stomatological Hospital, School of Stomatology, Capital Medical University, Beijing, China; 3 Department of General Dentistry and Integrated Emergency Dental Care, Capital Medical University School of Stomatology, Beijing, 100050 China; 4 Beijing Laboratory of Oral Health, Capital Medical University, Beijing, China; 5 Research Unit of Tooth Development and Regeneration, Chinese Academy of Medical Sciences, Beijing, China

**Keywords:** lncRNA SNHG1, Human jaw bone marrow mesenchymal stem cells, Chondrogenic differentiation, Mitochondria

## Abstract

**Background:**

Cartilage is a kind of avascular tissue, and it is difficult to repair itself when it is damaged. In this study, we investigated the regulation of chondrogenic differentiation and vascular formation in human jaw bone marrow mesenchymal stem cells (h-JBMMSCs) by the long-chain noncoding RNA small nucleolar RNA host gene 1 (SNHG1) during cartilage tissue regeneration.

**Methods:**

JBMMSCs were isolated from the jaws via the adherent method. The effects of lncRNA SNHG1 on the chondrogenic differentiation of JBMMSCs in vitro were detected by real-time fluorescence quantitative polymerase chain reaction (RT-qPCR), Pellet experiment, Alcian blue staining, Masson’s trichrome staining, and modified Sirius red staining. RT-qPCR, matrix gel tube formation, and coculture experiments were used to determine the effect of lncRNA SNHG1 on the angiogenesis in JBMMSCs in vitro. A model of knee cartilage defects in New Zealand rabbits and a model of subcutaneous matrix rubber suppositories in nude mice were constructed for in vivo experiments. Changes in mitochondrial function were detected via RT-qPCR, dihydroethidium (DHE) staining, MitoSOX staining, tetramethyl rhodamine methyl ester (TMRM) staining, and adenosine triphosphate (ATP) detection. Western blotting was used to detect the phosphorylation level of signal transducer and activator of transcription 3 (STAT3).

**Results:**

Alcian blue staining, Masson’s trichrome staining, and modified Sirius Red staining showed that lncRNA SNHG1 promoted chondrogenic differentiation. The lncRNA SNHG1 promoted angiogenesis in vitro and the formation of microvessels in vivo. The lncRNA SNHG1 promoted the repair and regeneration of rabbit knee cartilage tissue. Western blot and alcian blue staining showed that the JAK inhibitor reduced the increase of STAT3 phosphorylation level and staining deepening caused by SNHG1. Mitochondrial correlation analysis revealed that the lncRNA SNHG1 led to a decrease in reactive oxygen species (ROS) levels, an increase in mitochondrial membrane potential and an increase in ATP levels. Alcian blue staining showed that the ROS inhibitor significantly alleviated the decrease in blue fluorescence caused by SNHG1 knockdown.

**Conclusions:**

The lncRNA SNHG1 promotes chondrogenic differentiation and angiogenesis of JBMMSCs. The lncRNA SNHG1 regulates the phosphorylation of STAT3, reduces the level of ROS, regulates mitochondrial energy metabolism, and ultimately promotes cartilage regeneration.

**Supplementary Information:**

The online version contains supplementary material available at 10.1186/s13287-024-03793-2.

## Background

Human activities are inseparable from joint movement, and articular cartilage plays a role in relieving stress and lubricating joints. Once the cartilage is damaged or degenerated [1, 2], it is difficult to repair itself, because the main components of articular cartilage are collagen fibers and proteoglycans in addition to water, which lack a blood supply and cells cannot migrate to the defect site [3]. At present, the surgical treatment of cartilage defects mainly involves the direct use of growth factors, autologous transplantation, or allogeneic transplantation [4], but there are several problems, such as limited donor sites and immune responses [5]. In addition, synthetic cartilage scaffolds have attracted widespread attention because they are not limited by supply and can be functionalized [6, 7]. However, after implantation, it easily decomposes under joint force, and the production of hyaline cartilage is inefficient and inefficient [8–10].

Mesenchymal stem cells (MSCs) are considered an ideal choice for cartilage regeneration because they can differentiate into chondrocytes with biological functions [11]. In addition, the nutrients in articular cartilage are mainly provided by synovial fluid and subchondral blood vessels. Tissue repair includes inflammation, proliferation, and remodeling [12, 13], and the proliferation stage includes angiogenesis [14, 15]. MSCs not only undergo differentiation but also release cytokines via paracrine signaling to activate dermal fibroblasts, promote the production of collagen, and promote angiogenesis, thus promoting tissue healing [16]. There is evidence that intra-articular or intravascular injection of stem cells is effective [17]. Bone marrow mesenchymal stem cells (BMSCs) can promote osteogenesis, chondrogenesis, and angiogenesis in vivo, and a sufficient number of BMSCs are easily obtained; therefore, they are widely studied [18]. At present, studies have confirmed that BMSCs can be used for cartilage regeneration. BMSCs can not only inhibit the focal death of cartilage through derived exosomes [19] but can also be combined with hydrogels to differentiate cartilage in vivo and in vitro to form functional chondrocytes [20], which is helpful for cartilage regeneration. Dorotka et al. [21, 22] established sheep cartilage defect models and implanted BMSCs to treat articular cartilage defects. The results confirmed the cartilage regeneration potential of BMSCs. In addition, BMSCs are beneficial for cartilage reconstruction in vivo and in vitro studies of temporomandibular joint osteoarthritis (TMJOA) [23]. In addition, BMSCs secrete angiogenic factors, which are potential regulators of angiogenesis, to promote the proliferation, migration, angiogenesis, and tissue repair of human umbilical vein endothelial cells (HUVECs) [24]. However, BMSC transplantation also has some problems, such as the efficiency of direct differentiation of MSCs into specific cell types.

Long noncoding RNAs (lncRNAs) are a class of noncoding RNAs with a length of more than 200 nucleotides [25] that play crucial roles in maintaining stem cell pluripotency, guiding stem cell differentiation, and preserving cartilage homeostasis [26–28]. Nguyen et al. [29] identified 230 lncRNAs related to chondrogenic differentiation, so lncRNAs also play a regulatory role in BMSC chondrogenic differentiation. For example, the lncRNA AC006064.4-201 is downregulated in aging and degenerated human cartilage and can destroy the stability of cyclin dependent kinase inhibitor 1B (CDKN1B) mRNA through interaction with polypyrimidine tract binding protein 1 (PTBP1), alleviate cartilage aging and prevent osteoarthritis [30]. LncRNA-CRNDE regulates BMSC chondrogenic differentiation through silent information regulator 1 (SIRT1)/sex-determining region Y box protein 9 (SOX9) and promotes cartilage regeneration [31]. In addition, the lncRNA DANCR upregulates the expression of Smad3 and signal transducer and activator of transcription 3 (STAT3) and promotes cartilage formation in synovial mesenchymal stem cells (SMSCs) [32]. The lncRNA ZNF667-AS1 protects cartilage in rheumatoid arthritis by regulating the janus kinase (JAK) / signal transducer and activator of transcription (STAT) pathway [33]. The lncRNA small nucleolar RNA host gene 1 (SNHG1) is a ncRNA located on chromosome 11 [34, 35]. Some studies have confirmed that the lncRNA SNHG1 inhibits osteogenic differentiation and promotes angiogenesis, but its role in chondrogenic differentiation is still unclear. The regulation of the JAK/STAT pathway by lncRNAs is related to cartilage regeneration. The lncRNA SNHG1 has a certain regulatory effect on the JAK/STAT pathway. In ligament fibroblasts, the lncRNA SNHG1 upregulates interferon gamma receptor 1 (IFNGR1) by sponging miR-320b and activating JAK/STAT signal transduction [36]. In addition, previous studies have shown that there is a positive correlation between lncRNA SNHG1 and methyltransferase like 3 (Mettl3) [37], and Mettl3-mediated m6A regulates SOX9 mRNA in the 3'UTR, promotes SOX9 translation, and promotes BMSC cartilage differentiation [38]. Therefore, it is speculated that the lncRNA SNHG1 has a regulatory effect on BMSC chondrogenic differentiation.

Mitochondria are the main sites for ATP production in animal and plant cells and are important organelles for promoting cell energy conversion. Stem cell differentiation is regulated by energy metabolism, so mitochondria play a certain role in regulating osteochondral regeneration. For example, Sirtuin 3 improves bone regeneration by regulating mitochondrial oxidative stress [39]. Moreover, the effective recovery of mitochondrial dysfunction enhances the migration of chondrocytes and significantly promotes cartilage regeneration [40]. The JAK/STAT pathway has a certain regulatory effect on mitochondria. For example, edaravone can improve mitochondrial damage through JAK/STAT signaling and protect against kidney damage caused by ischemia/reperfusion [41]. The analysis of transcription and phosphorylation protein omics of rheumatoid arthritis showed that the change in mitochondrial function was related to the increase in epidermal growth factor receptor (EGFR)-JAK-STAT3 signal transduction [42].

Therefore, we studied the effects of the lncRNA SNHG1 on angiogenesis and chondrogenic differentiation. Moreover, the regulatory mechanism of the lncRNA SNHG1 in cartilage regeneration through JAK/STAT and mitochondria and its ability to promote cartilage regeneration were investigated.

## Methods

### Cells and cell culture

All the experimental procedures were approved by the Ethics Committee of the Beijing Stomatological Hospital of the Capital Medical University (License No: CMUSH-IRB-KJ-PJ-2023-50). In this study, primary cells were isolated and cultured via a monolayer culture method. Jaw fragments were cleaned with sterile PBS (Biosharp, China), and cancellous bone, and bone marrow cavities were scraped into PBS and then centrifuged in 15 ml polypropylene conical tube at 1100 rpm for 6 min. Then cultured in mesenchymal stem cell medium (MSCM; ScienCell, Carlsbad, CA, USA).

### Lentivirus-mediated cell transfection

The cDNA of the human lncRNA SNHG1 and short hairpin RNAs (shRNAs) were inserted into the LV6 lentiviral vector (Genepharma, Suzhou, China) and GV112 lentiviral vector (Genechem, Shanghai, China), respectively. For lentivirus infection, human jaw bone marrow mesenchymal stem cells (h-JBMMSCs) were seeded in 100 mm culture dishes and cultured overnight to 50–60% confluence. The fresh medium MSCM was replaced after 12 h, and puromycin (2 μg/mL) was used for screening at 48 h after infection. The target sequences were as follows: lncRNA SNHG1 shRNA (sh-SNHG1), 5′-GGTTTCAAGGCCATAGCTTTA-3′; and control shRNA (sh-Control), 5′-TTCTCCGAACGTGTCACGT-3′. The primer sequences are shown in Supplementary Table 1.

### In vitro chondrogenic differentiation

H-JBMMSCs were cultured in 6/12-well plate chondrogenic-induced conditioned medium. It is mainly composed of DMEM (Gibco, USA), 50 µg/mL ascorbic acid (Sigma, USA), 100 μg/mL sodium pyruvate (Sigma, USA), 40 μg/mL L-proline (Sigma, USA), 10 ng/mL transforming growth factor-β (TGF-β1, Sigma, USA), insulin-transferrin-selenium (ITS, ThermoFisher, USA), and 0.1 μM dexamethasone (Sigma, USA) [43]. After 0 and 7 days of chondrogenic induction of h-JBMMSCs, the expression of the lncRNA SNHG1 was detected by RT-qPCR. To further explore the effect of lncRNA SNHG1 on the chondrogenic differentiation of h-JBMMSCs, h-JBMMSCs were divided into sh-Control, sh-SNHG1, Vector, and SNHG1 groups to induce chondrogenic differentiation in vitro. After 0, 1, or 2 weeks of chondrogenic differentiation, RNA was collected to detect the mRNA levels of cartilage-related factors. The primer sequences are shown in Supplementary Material 1. After 3 weeks of induction, Alcian blue staining was performed according to the instructions. After drying the plate, 300 μl of 6 M guanidine hydrochloride (GuHCl, Sigma, USA) was added to dissolve the stain for 12 h, and the OD value was measured by an enzyme-labeled instrument at 620 nm for quantitative analysis. The 1 mM reactive oxygen species (ROS) inhibitor N-acetylcysteine (NAC, MCE, USA) and the concentration gradient JAK inhibitor (iJAK, MCE, USA) tofacitinib citrate were added at the same time during each fluid change. RNA or protein was collected for follow-up detection after 1 week of chondrogenic induction, and Alcian blue staining was performed after 3 weeks.

### Pellet experiment and histological examination

The cells with good growth status were digested, centrifuged in 15 ml polypropylene conical tube at 1100 rpm for 6 min and then washed with PBS again. The total number of cells was 4–5 × 10^5^/tube, and each tube contained 500 μl of chondrogenic medium. After centrifuged in 15 ml polypropylene conical tube at 1100 rpm for 6 min, the bottle cap was unscrewed and placed in a cell incubator at 37 °C and 5% CO_2_. After 4 weeks of culture, alcian blue staining (Solarbio, China), Masson’s trichrome staining (Solarbio, China), and modified Sirius Red staining (Solarbio, China) were performed according to the instructions.

### In vitro tube formation and coculture experiments

The Matrigel matrix (Corning, USA) was stored in a refrigerator at 4 °C overnight and was dissolved in liquid before use. The whole experiment was carried out on ice. Before the experiment, the 96-well plate, ice box, and sterile gun head were placed in a refrigerator for precooling. A 96-well plate was placed on an ice box, and 50 μl of Matrigel was added to each well in the center of the bottom of the vertical well, with 3 wells in each group. After incubation for 30 min to 1 h in a cell incubator at 37 °C, the h-JBMMSCs were still divided into four groups: sh-Control, sh-SNHG1, Vector, and SNHG1. After being mixed with HUVECs at a ratio of 1:1, the cells were inoculated at a ratio of 2 × 10^4^/well and 100 μl/well. The cells were cultured in a cell incubator for approximately 4–6 h, and then observed under an inverted biological microscope (Olympus, Japan) at 10× magnification. Branches were not dyed with calcien AM, three fields of view were randomly selected from each well for ImageJ analysis of branch number or branch length. The number of cells in the upper and lower layers of the coculture chamber was 2 × 10^5^/well, with 3 wells in each group. HUVECs were inoculated in the lower layer and h-JBMMSCs were inoculated in the upper layer. After 3 days of coculture, HUVECs RNA was extracted to detect the expression of vascular-related factors. The primer sequences are shown in Supplementary Material 1.

### Establishment of rabbit cartilage defect model and histological examination

The animal experiment was approved by the Animal Ethics Committee of the Beijing Stomatological Hospital, Capital Medical University (KQYY-202303-001). If these animals underwent successful surgery, they were included in the study, and if they died prematurely, they were excluded. According to the calculation formula of sample size, it is assumed that alpha = 0.05 and power = 0.80 are used for calculation. Nine male New Zealand rabbits (6 months old, 2.5–3.0 kg) were randomly divided into the Sham, Vector, and SNHG1 groups (n = 6). Random numbers were generated using the standard = RAND () function in Microsoft Excel. The random allocator did not know the actual grouping, and the surgical operator and the data analyst did not know each other about the animal grouping. All animals were maintained on a normal day and night light cycle and were allowed to eat and drink at will. After anesthesia with 3% pentobarbital sodium (35 mg/kg), both knees were skinned and disinfected. The knee was opened via the medial patellar approach, and the soft tissue was removed to expose the knee joint. In the Sham group, only the joint capsule was opened to expose the knee joint without any injury. In the Vector and SNHG1 groups, the knee joint was exposed, and a round osteochondral defect with a diameter of 4 mm and a depth of 2 mm was made in the medial femoral condyle. After washing to stop the bleeding, 50 μl of Matrigel matrix containing 1 × 10^6^ cells were added to the defect. The plates were sutured layer by layer, bandaged and fixed. Each rabbit was injected with penicillin (100,000 U/kg) for 7 days to prevent infection. Health status is monitored through the assessment of body, food and water intake and animal activities. Three months after the operation, all rabbits were euthanized by intravenous injection of 3% pentobarbital sodium (100 mg/kg). The morphology of cartilage regeneration was graded by five raters who were unaware of the experiment according to the International Cartilage Repair Society (ICRS) scoring system. In addition to HE staining, Alcian blue staining, Masson’s trichrome staining, and Safranin O/Fast green staining were performed according to the instructions.

### In vivo tube-forming experiment

Eight-week-old female nude mice were selected for in vivo tube-forming experiments and divided into the sh-Control group, sh-SNHG1 group, Vector group, and SNHG1 group, with 5 mice in each group (n = 5). The cells of h-JBMMSCs and HUVECs were digested and counted respectively, and made into cell suspensions. 1 × 10^6^ cells of each cell were mixed with 100–150 μl of Matrigel matrix, a 1 mL needle was used to subcutaneously inject the cells into the back, and samples were taken 14 days later. The nude mice were euthanized by cervical dislocation after inhaling isoflurane anesthesia. After sampling, the samples were subjected to hematoxylin–eosin (HE) staining, immunohistochemistry (IHC) staining, and immunofluorescence (IF) staining.

### Immunohistochemistry and immunofluorescence staining

The paraffin sections were repaired in sodium citrate antigen repair buffer at 95 °C for 15 min. After washing with PBS, an appropriate amount of endogenous peroxidase blocking agent was added, and the sections were incubated in the dark for 10 min. CD31 primary antibody (Bioss, Beijing, China) from the rabbit was added, and the sections were incubated at 4 °C overnight. Then, the reaction enhancer was decreased, and the sections were incubated at 37 °C for 20 min. The appropriate amount of goat anti-mouse/IgG-free polymer (ZSGB-BIO, Beijing, China) labeled with enhanced enzyme was added dropwise for IHC staining, and green fluorescent secondary antibody (1:500, Life Technologies, US) was added dropwise for IF staining, and the sections were incubated at 37 °C in the dark for 20 min. IF staining was blocked with an anti-fluorescence quencher containing DAPI, and IHC staining was performed with DAB chromogenic solution. Then, the sections were dehydrated, cleared, and sealed with neutral gum. ImageJ software was used to analyze the results.

### Cytosolic ROS and MitoSOX RED staining of cells

The dihydroethidium (DHE) probe (Applygen, China) was diluted to 5 μM in serum-free medium, and the cells were incubated at 37 °C for approximately 30 min. At an excitation wavelength of 594 nm, a field of view was randomly selected for imaging, and the fluorescence intensity was subsequently used for quantitative analysis. The MitoSOX Red probe is permeable to the cell membrane, and it emits red fluorescence after entering mitochondria. This fluorescence is used to measure the level of mitochondrial ROS (mROS). The 10 μM MitoSOX Red working solution (MCE, USA) was prepared in serum-free medium, incubated in the dark at 37 °C for 30 min, and then photographed and analyzed.

### Detection of mitochondrial membrane potential and ATP

The stock solution was diluted with serum-free cell culture medium to obtain a 1 μM TMRM working solution (MCE, USA), which was incubated in the dark for 30 min. Then, the dye working solution was added, and the culture medium was washed twice for 5 min each time. The cells were observed under an inverted fluorescence phase contrast microscope (BZ-X800, Keyence, China) at 40× magnification, and randomly selected fields of vision were used to obtain images for statistical analysis. Then, 100 μl of ATP detection working solution (Beyotime Biotechnology, China) was added to each well of a black 96-well plate, and the plate was allowed to stand for 3–5 min. Then, 20 μl of sample was added to the wells, and the RLU was measured at a wavelength of 562 nm.

### Reverse transcription-quantitative polymerase chain reaction (RT-qPCR)

Total RNA was extracted by using the TRIzol reagent (Invitrogen, USA). Approximately 1 μg aliquots of RNA were reverse transcribed into cDNA (Invitrogen, USA). A QuantiTect SYBR Green PCR kit (Qiagen, Hilden, Germany) and an Archimed-X6 real-time PCR detection system (Rocgene, Beijing, China) were used for relative quantitative PCR. The relative expression levels of SNHG1 were normalized to those of GAPDH. The data were analyzed by the 2^−△△^CT method.

### Western blot analysis

RIPA buffer (ThermoFisher, USA) supplemented with protease inhibitor cocktail (PIC, Cell Signaling Technology, USA) and phenylmethanesulfonyl fluoride (PMSF, Shanghai Aladdin Biochemical Technology, China) was used to extract proteins. Protein samples were blocked for 1 h after gel electrophoresis and membrane transfer. The primary antibody was incubated overnight at 4 °C, and the secondary antibody was incubated at room temperature for 1 h. The samples were observed with a Super ECL Plus kit (Applygen, Beijing, China). The primary antibodies used were as follows: STAT3 (1: 1000, ab119352, Abcam, USA), p-STAT3 (1: 1000, ab32143, Abcam, USA), and GAPDH (Cat No. G8795, Sigma-Aldrich, USA).

### Statistical analysis

Statistically significant differences (*P* < 0.05) were determined using the Student’s t test. GraphPad Prism 8 software was used for all the statistical analyses.

## Results

### SNHG1 overexpression enhanced chondrogenic differentiation in h-JBMMSCs

**Fig. 1:**
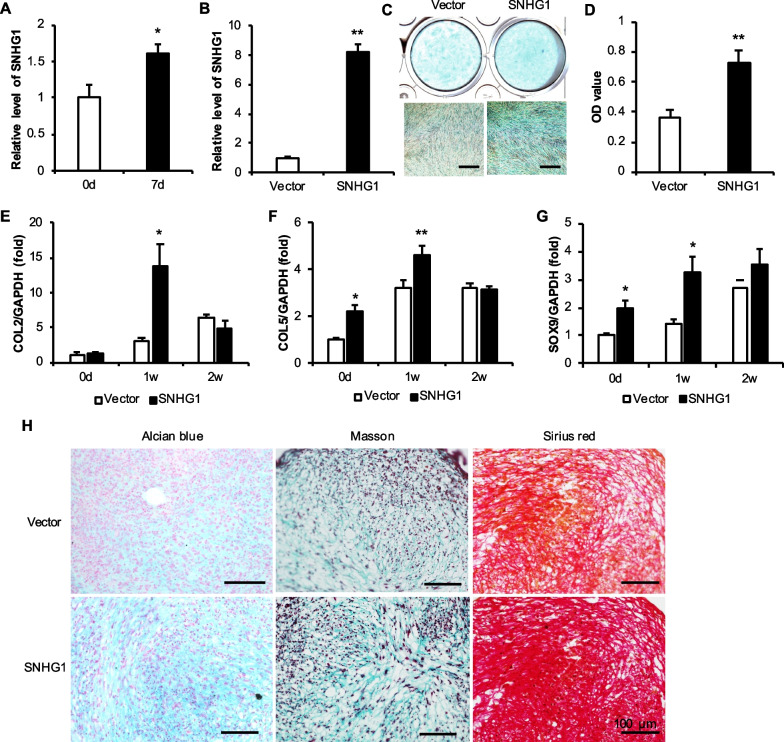
Effect of lncRNA SNHG1 overexpression on the chondrogenic ability of h-JBMMSCs. **A** The mRNA level of the lncRNA SNHG1 gene at 0 and 7 days after h-JBMMSCs chondrogenic induction; **B** RT-qPCR detection of h-JBMMSCs overexpressing the lncRNA SNHG1 gene; **C**–**D** After 3 weeks of chondrogenic induction after h-JBMMSCs overexpressing the lncRNA SNHG1, Alcian blue staining and quantitative analysis were carried out; **E**–**G** The mRNA levels of cartilage-related factors after 0, 1 and 2 weeks of chondrogenic induction after overexpression of the lncRNA SNHG1 by h-JBMMSCs; **H** After h-JBMMSCs overexpressing the lncRNA SNHG1, Pellet chondrocytes were stained with Alcian blue, Masson trichrome and modified Sirius red. The scale is 100 μm. Statistical analysis was performed by Student’s t test. Bar graphs: Data are presented as the mean ± SD. ^*^P < 0.05 and ^**^P < 0.01

RNA was collected after 0 and 7 days of chondrogenic differentiation of h-JBMMSCs, and PCR revealed that the expression of the lncRNA SNHG1 increased significantly after 7 days of chondrogenic induction (Fig. 1A). Moreover, the transfection efficiency of the lncRNA SNHG1-overexpressing virus in h-JBMMSCs was verified (Fig. 1B). After 0, 1, and 2 weeks of chondrogenic induction, the expression of collagen II (COL2), collagen V (COL5), and SOX9 in the SNHG1 group increased significantly at 1 w (Fig. 1E–G). But at 2 weeks, there is no difference at 2w between the groups in COL2, COL5, and SOX9 expression. Each stage of chondrogenic differentiation of MSCs is controlled by different transcription factors (TF). TF regulates the gene expression level, and these cartilage-related indexes change dynamically during the whole process [44, 45]. In addition, we studied by intervening gene expression, but the influence of genes on cartilage differentiation showed different trends at different time points, and there may be no obvious difference at some time points. In addition, after 3 weeks of induction, compared with that of the Vector group, the staining of the SNHG1 group was deeper, the OD value was significantly greater, and the expression of sulfate mucin in the SNHG1 group increased (Fig. 1C–D). Alcian blue was used to stain glycoproteins modified by chondroitin sulfate and keratin sulfate, indicating that the expression of sulfate mucin in the SNHG1 group was increased. The pellet cartilage ball experiment showed that compared with those in the Vector group, Masson staining, Alcian blue staining, and Sirius red staining were more intense in the SNHG1 group (Fig. 1H). The results showed that the increased expression of the lncRNA SNHG1 in h-JBMMSCs promoted chondrogenic differentiation in vitro.

### SNHG1 knockdown inhibited the chondrogenic differentiation in h-JBMMSCs

**Fig. 2:**
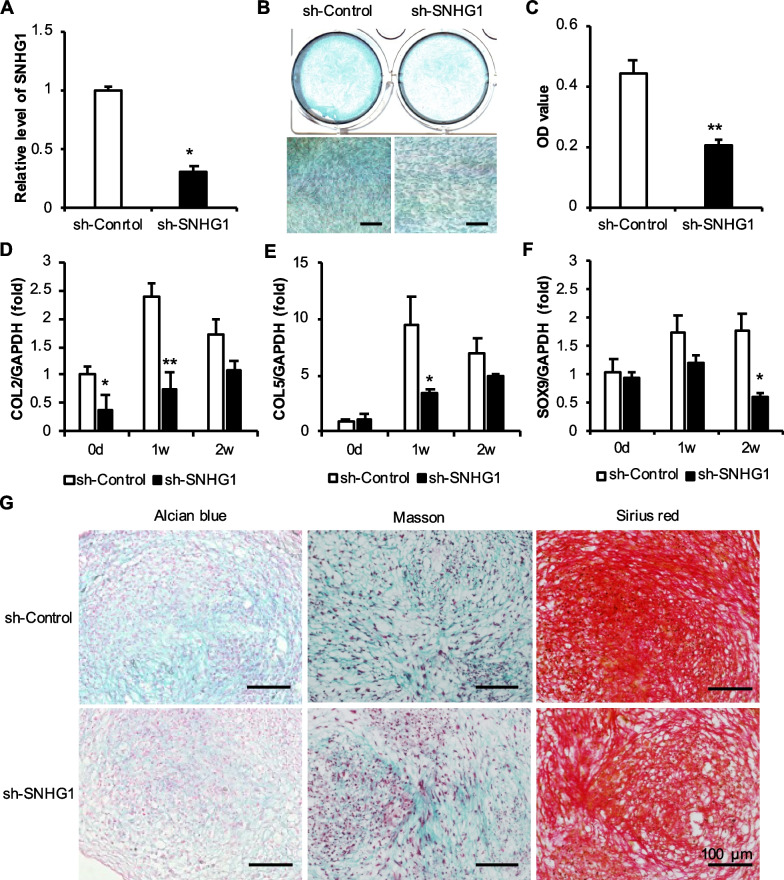
Effect of lncRNA SNHG1 knockdown on the chondrogenic ability of h-JBMMSCs. **A** RT-qPCR detection of h-JBMMSCs with lncRNA SNHG1 gene knockdown; **B**–**C** Alcian blue staining and quantitative analysis were performed after chondrogenic induction for 3 weeks after h-JBMMSCs knocked down lncRNA SNHG1; **D**–**F** The mRNA level of cartilage-related factors was 0, 1 and 2 weeks after chondrogenic induction after knocking down lncRNA SNHG1 by h-JBMMSCs; **G** Alcian blue staining, Masson trichrome staining and modified Sirius red staining of Pellet chondrocytes after h-JBMMSCs knocked down lncRNA SNHG1. The scale is 100 μm. Statistical analysis was performed by Student’s t test. Bar graphs: Data are presented as the mean ± SD. ^*^P < 0.05 and ^**^P < 0.01

PCR revealed that the expression of the lncRNA SNHG1 in sh-SNHG1 cells was significantly reduced (Fig. 2A). After 0, 1, and 2 weeks of chondrogenesis in the sh-Control group and sh-SNHG1 group, the expressions of COL2, COL5, and SOX9 in the sh-SNHG1 group decreased at 1 week and 2 weeks, among which the expression of COL2 and COL5 decreased significantly at 1 week and that of SOX9 decreased significantly at 2 weeks (Fig. 2D–F). In addition, after 3 weeks of induction, the sh-SNHG1 group exhibited less intense staining than the sh-Control group, and the quantitative analysis revealed that the OD value was significantly lower (Fig. 2B–C). Four weeks after Pellet chondrocytes were induced, paraffin section staining showed that the sulfate mucin in the sh-SNHG1 group was lighter blue, the collagen fibers in Masson staining were lighter green, and the collagen fibers in Sirius red staining were lighter red (Fig. 2G). The results showed that the decreased expression of the lncRNA SNHG1 in h-JBMMSCs inhibited the formation of sulfated mucin and collagen fibers, and inhibited chondrogenic differentiation in vitro.

### JBMMSCs overexpressed SNHG1 to promote angiogenesis and tube formation

**Fig. 3:**
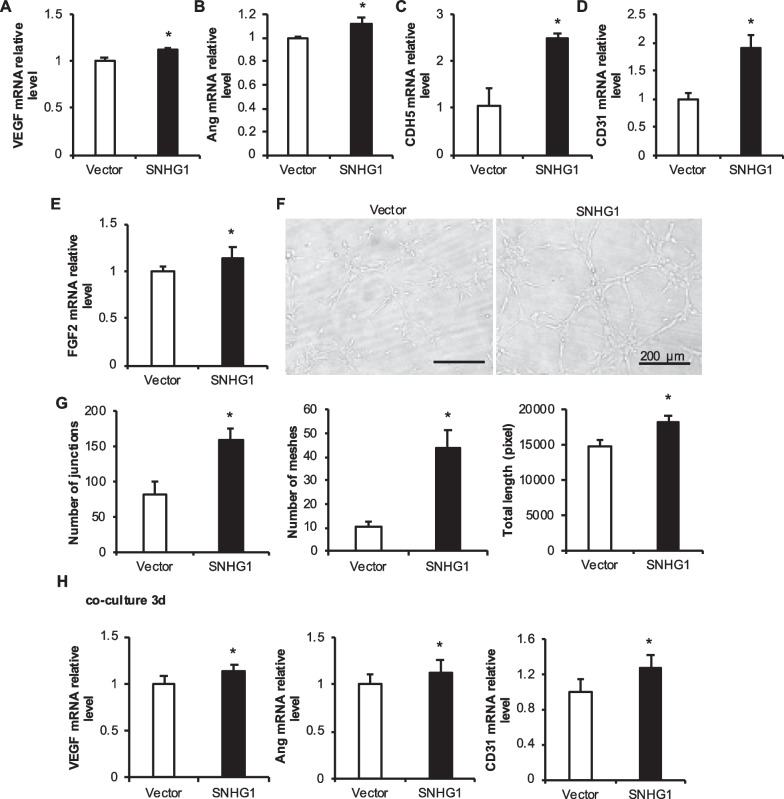
Effect of the overexpression of the lncRNA SNHG1 by JBMMSCs on the angiogenesis of HUVECs. **A**–**E** mRNA expression levels of angiogenesis-related factors in JBMMSCs overexpressing the lncRNA SNHG1; **F**–**G** Angiogenic structure diagram and statistical analysis of the number of pore structures; **H** mRNA expression of angiogenesis-related factors in HUVECs cocultured with JBMMSCs overexpressing the lncRNA SNHG1. The scale is 100 μm. Statistical analysis was performed by Student’s t test. Bar graphs: Data are presented as the mean ± SD. ^*^P < 0.05 and ^**^P < 0.01

After overexpressing the lncRNA SNHG1, the expression of angiogenesis-related factors in h-JBMMSCs was detected. The mRNA expression levels of vascular endothelial growth factor (VEGF), angiopoietin (Ang), VE-cadherin (CDH5), CD31, and fibroblast growth factor-basic (FGF-2) were increased in the SNHG1 group (Fig. 3A–E). In addition, Vector and SNHG1 transfected h-JBMMSCs were cocultured with HUVECs for 3 days, after which the RNA was collected from the HUVECs. The mRNA levels of VEGF, Ang, and CD31 in HUVECs increased significantly (Fig. 3H). Vector and SNHG1 cells were mixed with HUVECs cells at a 1: 1 ratio and then inoculated on solidified matrix glue. After 4 h, the tube-forming effect was observed under a microscope and statistically analyzed by ImageJ software. The number of junctions, the number of meshes, and the total length of tubes in the SNHG1 group increased significantly (Fig. 3F–G). These results show that the overexpression of the lncRNA SNHG1 affects the expression of the tube-forming factors of h-JBMMSCs and HUVECs to some extent, thus enhancing their tube-forming ability.

**Fig. 4:**
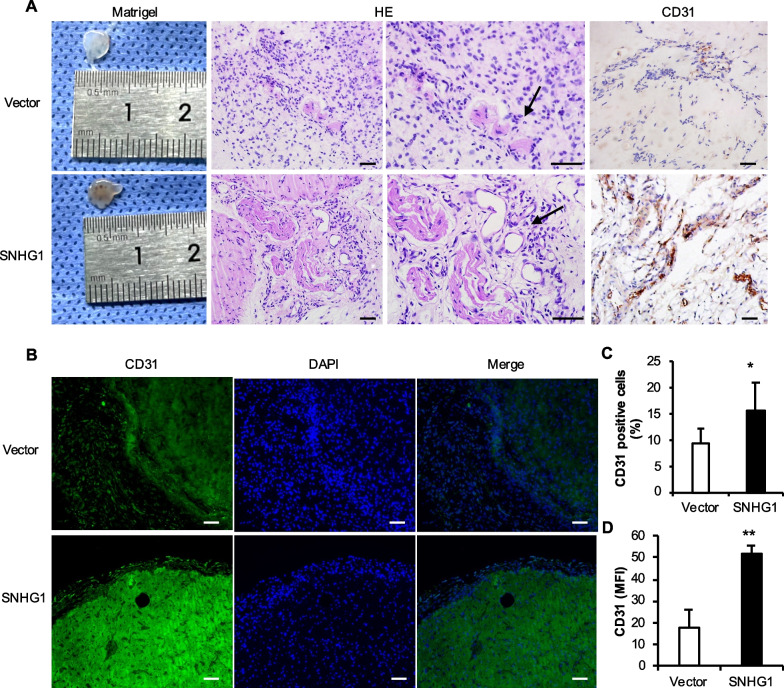
Effect of the overexpression of the lncRNA SNHG1 on the tube formation ability. **A** The general picture, HE staining and CD31 IHC staining of matrix rubber suppositories, with black arrows pointing to microvessels; **B** CD31 IF staining was used to detect the expression and quantification of CD31 protein after overexpression of lncRNA SNHG1; **C** Statistics of percentage of CD31-positive cells by IHC staining; **D** Statistics of average fluorescence intensity of CD31 IF staining. The scale is 50 μm. Statistical analysis was performed by Student’s t test. Bar graphs: Data are presented as the mean ± SD. ^*^P < 0.05 and ^**^P < 0.01

After 14 days of replanting the Matrigel matrix in nude mice, we found that compared with those in the Vector group, the matrix gel plug was filiform light red, and that in the SNHG1 group was red. In the SNHG1 group, HE staining revealed that many tubular structures were covered by pericyte-like cells, CD31 IHC staining revealed a dark yellow–brown color, and the percentage of positive cells increased significantly (Fig. 4A and C). CD31 IF staining revealed bright CD31-positive staining in the SNHG1 group. Quantitative analysis of IF data by ImageJ software revealed that the mean fluorescence intensity (MFI) of the SNHG1 group was significantly greater than that of the Vector group (Fig. 4B and D).

### SNHG1 knockdown in JBMMSCs inhibited angiogenesis and tube formation

**Fig. 5:**
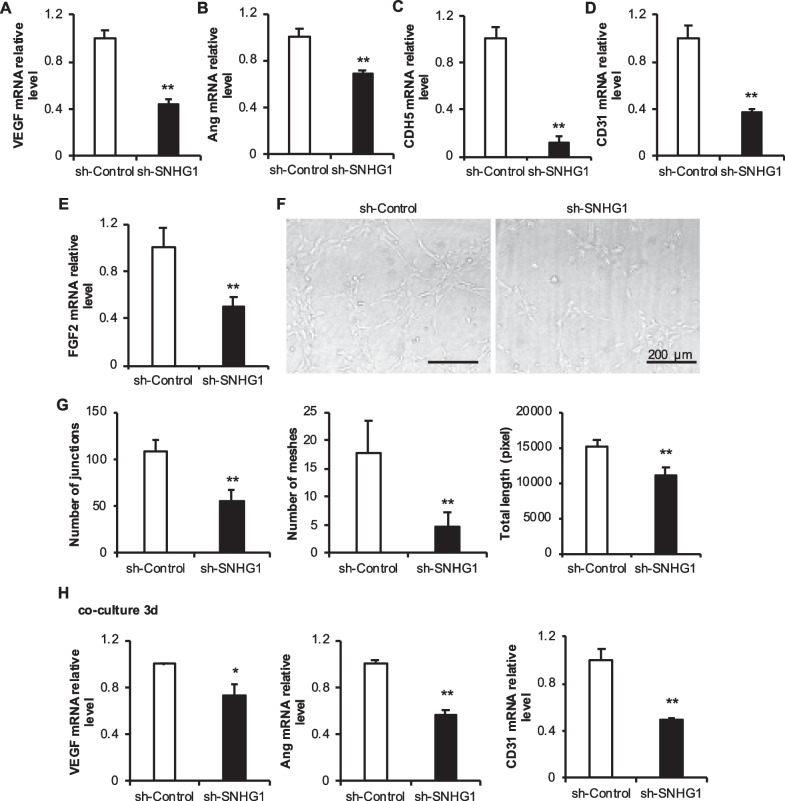
Effect of knocking down lncRNA SNHG1 by JBMMSCs on the angiogenesis ability of HUVECs. **A**–**E** mRNA expression levels of angiogenesis-related factors in JBMMSCs after knocking down lncRNA SNHG1; **F**–**G** Angiogenic structure diagram and statistical analysis of the number of pipe structures; **H** mRNA expression of angiogenesis-related factors in HUVECs cocultured with JBMMSCs after knocking down lncRNA SNHG1. The scale is 200 μm. Statistical analysis was performed by Student’s t test. Bar graphs: Data are presented as the mean ± SD. ^*^P < 0.05 and ^**^P < 0.01

Moreover, after knocking down lncRNA SNHG1, the mRNA levels of the angiogenesis-related factors VEGF, Ang, CDH5, CD31 and FGF-2 decreased significantly in the JBMMSCs (Fig. 5A–E). After 3 days of coculture with HUVECs, the mRNA levels of VEGF, Ang and CD31 in HUVECs also decreased significantly (Fig. 5H). The tube-forming ability was also obviously weakened (Fig. 5F–G).

**Fig. 6:**
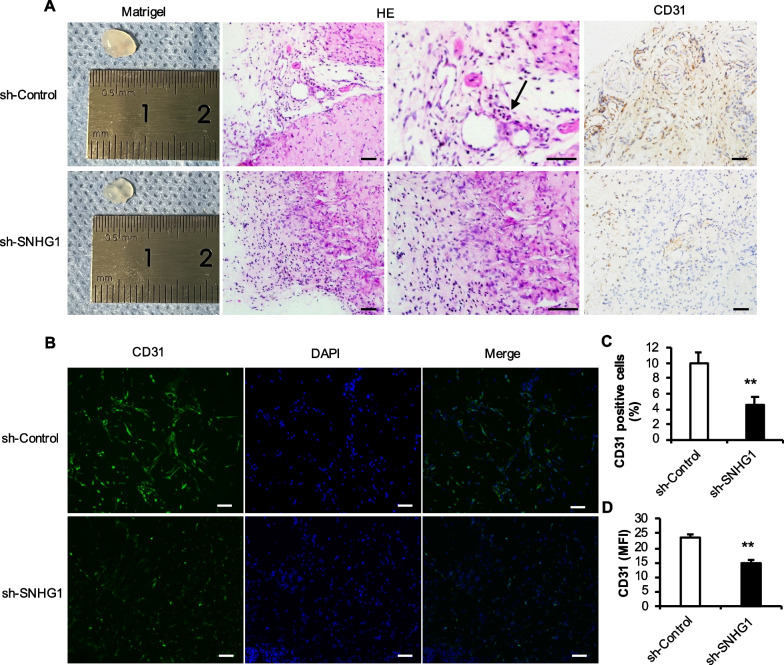
Effect of knocking down lncRNA SNHG1 on tube formation ability. **A** The general picture, HE staining and CD31 IHC staining of matrix rubber suppositories, with black arrows pointing to microvessels; **B** CD31 IF staining was used to detect the expression and quantification of CD31 protein after knocking down lncRNA SNHG1; **C** Statistics on the percentage of CD31 IHC-positive cells; **D** Statistics of average fluorescence intensity of CD31 IF staining. The scale is 50 μm. Statistical analysis was performed by Student’s t test. Bar graphs: Data are presented as the mean ± SD. ^*^P < 0.05 and ^**^P < 0.01

Moreover, after knocking down lncRNA SNHG1, compared with that in the sh-Control group, the Matrigel matrix was filiform light red, but it was almost transparent in the sh-SNHG1 group. HE staining revealed almost no lumen structure in the sh-SNHG1 group, and CD31 IHC staining revealed a light yellow-brown color, and the percentage of positive cells was significantly lower in the sh-SNHG1 group than in the sh-Control group (Fig. 6A and C). CD31 IF staining revealed that CD31-positive staining was not detected or rare in the sh-SNHG1 group. The quantitative analysis of IF by ImageJ software showed that the MFI of the sh-SNHG1 group was significantly lower than that of the sh-Control group (Fig. 6B and D).

### Overexpression of SNHG1 promoted cartilage regeneration in rabbits

**Fig. 7:**
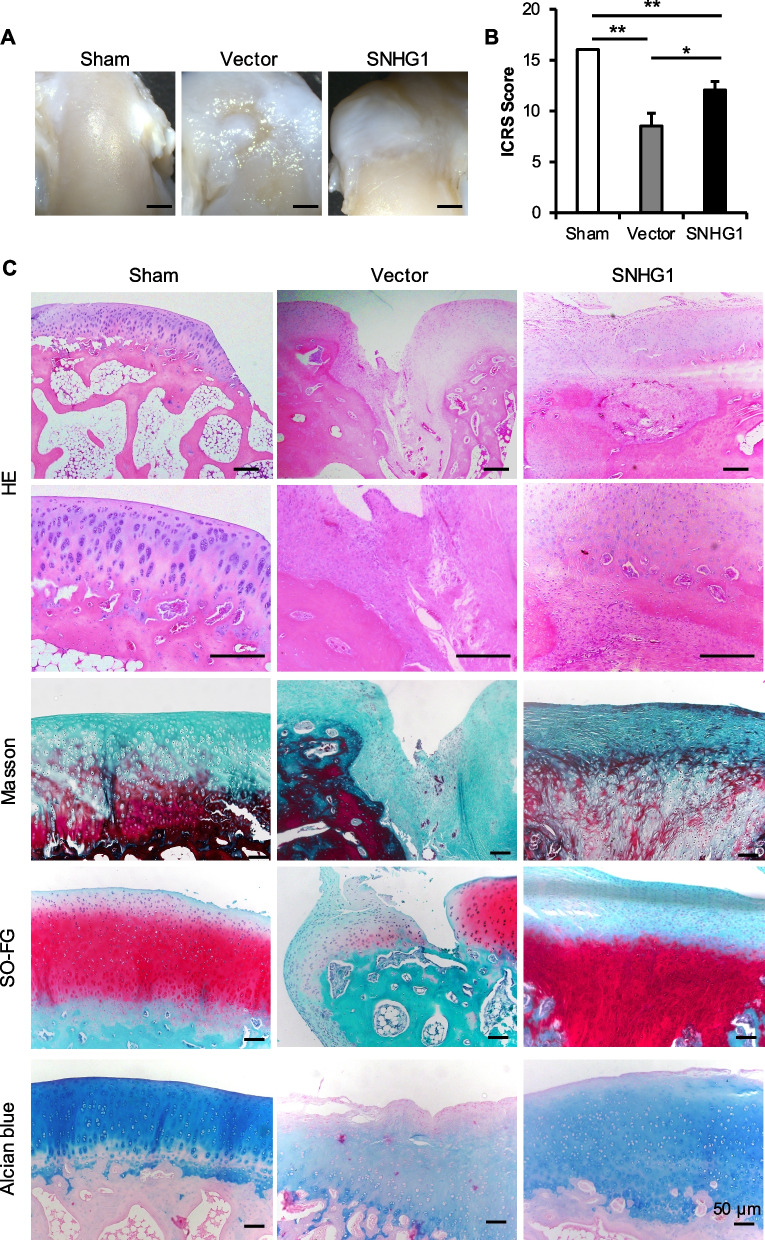
Effect of lncRNA SNHG1 overexpression on cartilage regeneration in vivo. **A**–**B** Gross view and ICRS score of cartilage injury repair 3 months after operation; **C** Pathological staining of cartilage injury and repair 3 months after operation, including HE staining, Masson staining, saffron O/Fast green staining and Alcian blue staining. The scale is 50 μm. Statistical analysis was performed by Student’s t test. Bar graphs: Data are presented as the mean ± SD. ^*^P < 0.05 and ^**^P < 0.01

To establish a model of knee cartilage injury in New Zealand rabbits, samples were taken 3 months after replanting the JBMMSCs. The ICRS score was calculated, and compared with that in the Vector group, the cell replanting in the SNHG1 group significantly improved the ICRS score, indicating that the overexpression of the lncRNA SNHG1 can repair the cartilage injury and result in a more complete and smoother surface (Fig. 7A–B). After a routine sectioning, HE staining revealed that the outermost perichondrium area in the Sham group was smooth and complete, and there was no obvious depression defect. The cartilage matrix was blue-purple due to the presence of a large amount of acidic mucopolysaccharide, and the cartilage pit was strongly purple due to chondroitin sulfate. Compared with those of the Vector group, the surface perichondrium of the SNHG1 group was more complete, the replanted cells were long and flat, and cartilage lacunae could be seen. During Masson staining, the collagen fibers were dyed green. Compared with the Vector group, the SNHG1 group was greener. In the saffron O/Fast green staining, saffron O showed red staining for proteoglycans in the cartilage matrix, and compared with the Vector group, the SNHG1 group had obviously red staining, and the SNHG1 group had obviously blue staining (Fig. 7C).

### The influence of SNHG1 on JAK/STAT in h-JBMMSCs

**Fig. 8:**
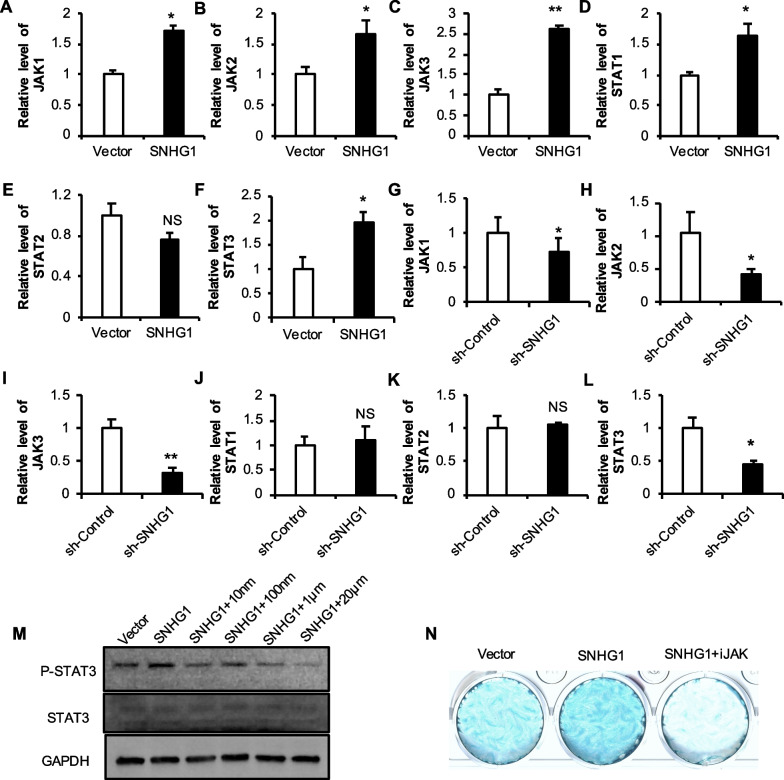
Effects of JAK on the chondrogenic differentiation of JBMMSCs. **A**–**L** The expression level of JAK/STAT mRNA after JBMMSCs were knocked down or overexpressing the lncRNA SNHG1; (M) WB was used to detect the protein expression level of p-STAT3/STAT3; (N) Alcian blue staining. Statistical analysis was performed by Student’s t test. Bar graphs: Data are presented as the mean ± SD. ^*^P < 0.05 and ^**^P < 0.01

After knockdown/overexpression, the expression levels of JAK/STAT mRNA in h-JBMMSCs were detected. Compared with that in the control group, the expression of JAK changed significantly after knocking down or overexpressing the lncRNA SNHG1. The expression of only STAT3 changed significantly after the knockdown or overexpression of the lncRNA SNHG1 (Fig. 8A–L), therefore, STAT3 was selected for further study. H-JBMMSCs were divided into Vector, SNHG1 and SNHG1 + iJAK groups (0, 10 nm, 100 nm, 1 μm, and 20 μm). After one week of chondrogenic induction, compared with that in the Vector group, the protein expression level of p-STAT3 in the SNHG1 group increased, while the usage of 10 nm, 1 μm, and 20 μm iJAK decreased significantly (Fig. 8M). It is speculated that JAK may affect chondrogenic differentiation by affecting the phosphorylation of STAT3.

To detect the effect of JAK on h-JBMMSCs, iJAK was added at the same time as chondrogenic induction, and after 3 weeks of induction, Alcian blue staining was performed. The overexpression of the lncRNA SNHG1 significantly promoted the chondrogenic differentiation of h-JBMMSCs, while the use of iJAK significantly inhibited the chondrogenic differentiation caused by the lncRNA SNHG1. These findings suggested that JAK may play a role in chondrogenic differentiation induced by the lncRNA SNHG1 (Fig. 8N).

### SNHG1 regulated h-JBMMSCs chondrogenic differentiation through mitochondrial energy metabolism

**Fig. 9:**
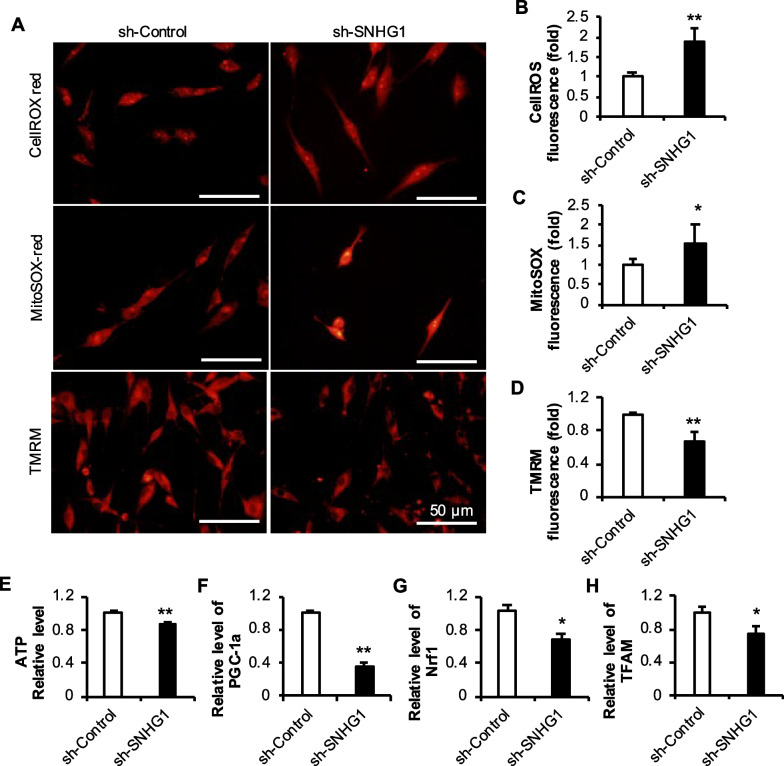
Effect of lncRNA SNHG1 knockdown on the mitochondrial function of JBMMSCs. **A**–**D** Cytoplasmic reactive oxygen species (cROS) and mitochondrial reactive oxygen species (mROS) levels and the mitochondrial membrane potential of JBMMSCs were quantified after lncRNA SNHG1 was knocked down; **E** ATP levels in JBMMSCs after lncRNA SNHG1 was knocked down; **F**–**H** Expression of mitochondrial biogenesis-related factors in JBMMSCs after lncRNA SNHG1 was knocked down. The scale is 50 μm. Statistical analysis was performed by Student’s t test. Bar graphs: Data are presented as the mean ± SD. ^*^P < 0.05 and ^**^P < 0.01

To detect the influence of decreased expression of the lncRNA SNHG1 on the levels of cytosolic reactive oxygen species (cROS) and mROS in JBMMSCs, the DHE red probe was used to measure the level of cROS, and the MitoSOX RED probe was used to measure the level of mROS. After the expression of the lncRNA SNHG1 decreased, the level of cROS in JBMMSCs increased significantly (Fig. 9A–B). ROS can induce mitochondrial damage, which further leads to increased ROS production in damaged mitochondria. Therefore, the MitoSOX RED probe was used to detect mROS, and compared with that in the sh-Control group, the level of mROS in the sh-SNHG1 group was significantly greater (Fig. 9A and C). Mitochondrial ROS can be released into the cytoplasm through mitochondrial permeability transition pores, which are also important sources of cROS in the cytoplasm.

The mitochondrial membrane potential (MMP, △ψm) is generated by the proton pump of the electron transfer chain, which is necessary for ATP production, so we used TMRM staining to evaluate mitochondrial membrane potential. The level of ATP is a marker of the level of mitochondrial energy metabolism. Compared with that in the sh-Control group, the fluorescence intensity of TMRM staining in the sh-SNHG1 group was decreased, indicating that the MMP was decreased (Fig. 9A–D). At the same time, a standard curve was drawn with different standard concentrations of the ATP detection kit, the OD values of the sh-Control and sh-SNHG1 groups were detected, and the corresponding ATP values were calculated from the standard curve. The ATP level in the sh-SNHG1 group was significantly lower than that in the sh-Control group (Fig. 9E). After knocking down the lncRNA SNHG1, the mRNA levels of mitochondrial biogenesis-related factors, such as peroxisome proliferator-activated receptor γ coactivator 1α (PGC-1α), nuclear respiratory factor (Nrf) 1 and mitochondrial transcription factor A (TFAM), decreased significantly (Fig. 9F–H).

**Fig. 10:**
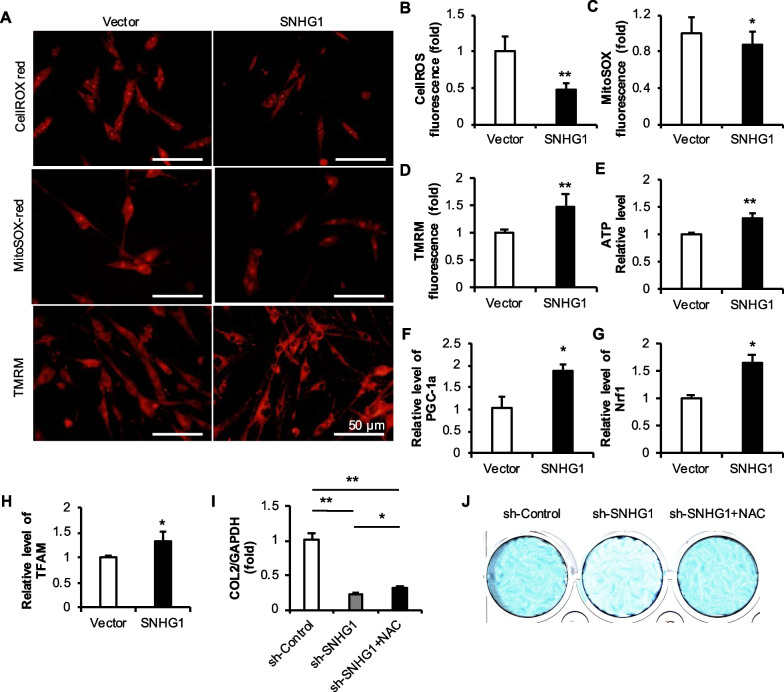
Effects of SNHG1 overexpression on mitochondrial function and the effects of ROS on chondrogenic differentiation. (**A**–**D**) Staining and quantitative analysis of cROS, mROS and mitochondrial membrane potential in JBMMSCs overexpressing the lncRNA SNHG1; **E** ATP levels in JBMMSCs overexpressing the lncRNA SNHG1; **F**–**H** Expression of mitochondrial biogenesis-related factors in JBMMSCs overexpressing the lncRNA SNHG1. **I** The mRNA level of COL2; **J** Alcian blue staining. The scale is 50 μm. Statistical analysis was performed by Student’s t test. Bar graphs: Data are presented as the mean ± SD. ^*^P < 0.05 and ^**^P < 0.01

After overexpressing the lncRNA SNHG1, changes in cROS and mROS levels were detected by the DHE red probe and MitoSOX RED probe. We found that the overexpression of the lncRNA SNHG1 significantly reduced the levels of cROS and mROS (Fig. 10A–C). TMRM staining showed that compared with that in the Vector group, the fluorescence intensity of TMRM in the SNHG1 group was greater, and the mitochondrial membrane potential was greater (Fig. 10A and D). At the same time, the OD values of the Vector and SNHG1 groups were detected by an ATP detection kit, and the corresponding ATP values were calculated. The ATP level in the SNHG1 group was significantly greater than that in the Vector group (Fig. 10E). After overexpressing the lncRNA SNHG1, the mRNA levels of PGC1-α, Nrf1, and TFAM increased significantly (Fig. 10F–H).

To further verify the effect of ROS levels on the chondrogenic regeneration of h-JBMMSCs, a rescue experiment was carried out with the ROS inhibitor NAC. H-JBMMSCs were divided into sh-Control, sh-SNHG1 and sh-SNHG1 + NAC groups. During chondrogenic induction, NAC was added at the same time while the medium was changed to a concentration of 1 mM. After one week of induction, PCR revealed that the addition of NAC alleviated the decrease in COL2 expression caused by the decrease in the expression of the lncRNA SNHG1 (Fig. 10  I) Alcian blue staining revealed that the decrease in the intensity of the blue signal caused by the decreased expression of the lncRNA SNHG1 was attenuated after NAC treatment, and the intensity of the Alcian blue staining increased (Fig. 10J).

## Discussion

The lncRNA SNHG1 was upregulated during the chondrogenic differentiation of h-JBMMSCs and promoted the expression of COL2, COL5, and SOX9. High expression of SOX9 promotes the expression of the extracellular matrix proteins aggrecan and COL2, which are unique to chondrocytes and constitute the main components of the extracellular matrix of hyaline cartilage [46]. In addition, the lncRNA SNHG1 promoted Alcian blue staining, Masson staining, and Sirius red staining in the chondrogenic induction of h-JBMMSCs in vitro. Masson’s trichrome and Sirius Red are both collagen dyes, which shows that the lncRNA SNHG1 promotes the formation of collagen fibers. The glycoproteins (PGs) in cartilage are mainly aggrecan, and the core of this protein is modified by glycosaminoglycan (GAG) side chains, which are mainly composed of chondroitin sulfate (CS) and keratin sulfate. Alcian blue can dye CS and keratin sulfate-modified glycoproteins. These results indicate that the lncRNA SNHG1 could promote chondrogenic differentiation of h-JBMMSCs in vitro.

In addition, we found that in h-JBMMSCs, the lncRNA SNHG1 can promote the expression of angiogenesis-related genes such as VEGF, Ang, and FGF2, suggesting that the lncRNA SNHG1 may have a regulatory effect on angiogenesis. VEGF is not only an essential factor for the formation of bone blood vessels but also a key factor for the survival of chondrocytes. Studies have shown that the lack of VEGF in mouse chondrocytes leads to massive cell death of articular chondrocytes [47]. FGF2 is a member of the FGF family [48]. In endothelial cells, FGF2 can not only induce angiogenesis but also regulate the expression of CDH5 and the formation of tight junctions, thus promoting angiogenesis [49, 50]. In addition, FGFs, which are abundant in the pericellular matrix of cartilage and bind to cell surface receptors, reduce the activity of aggrecanase, are helpful for cartilage repair [51]. In vitro, the lncRNA SNHG1 promoted the expression of tube-forming genes and promoted tube formation in HUVECs. Moreover, in vivo experiments revealed that the overexpression of the lncRNA SNHG1 also increased the number of microvessels and CD31 expression. CD31 is often used as a marker of vascular endothelium because it is highly expressed in all vascular endothelial cells in developing and mature individuals. These results indicate that the lncRNA SNHG1 has a certain angiogenic function.

The in vivo repair of cartilage defects indicated that the lncRNA SNHG1 promoted cartilage regeneration in h-JBMMSCs. Under normal circumstances, cartilage is an avascular tissue. When cartilage is damaged, if blood vessels invade cartilage, cartilage ossification is stimulated. Here, the lncRNA SNHG1 promoted angiogenesis and cartilage regeneration at the same time, presumably for the following reasons. Angiogenesis is the formation of new blood vessels from preexisting blood vessels, and BMSCs are nonhaematopoietic stem cells. At present, research on angiogenesis in BMSCs cannot be separated from that on angiogenesis in HUVECs, and the angiogenesis-promoting function of BMSCs mainly involves the secretion of cytokines or exosomes [52, 53]. The cartilage structure includes a calcified cartilage layer, which acts as a semipermeable membrane, allowing small molecules to pass through and form a barrier to maintain the stability of their respective microenvironment [54]. Therefore, the avascular characteristics of the cartilage itself and the barrier effect of the calcified cartilage layer weaken the ability of the lncRNA SNHG1 to promote the secretion of angiogenic factors in JBMMSCs. In addition, the formation of blood vessels is regulated by the oxygen content in the microenvironment. Although hypoxia can induce angiogenesis [55], sustained hypoxia of the same degree can inhibit angiogenesis [55], partly because the basement membrane fails to form around new blood vessel buds [56]. Therefore, although the lncRNA SNHG1 can promote the formation of HUVECs through h-JBMMSCs, it may not affect cartilage regeneration. In addition, the regulatory effect of the vascular system on the differentiation of stem cells is considered not only from the perspective of tube formation but also from the perspective of the joint effects of nutrient availability and osteochondral growth factor.

The JAK/STAT signal transduction pathway regulates a wide range of cellular processes, including cell growth, proliferation, differentiation, and apoptosis [57]. The lncRNA DANCR can regulate the regulatory axis of miR-1305-SMAD4 by upregulating the expression of Smad3 and STAT3 and promoting the chondrogenic differentiation of SMSCs [32]. A deficiency in the proteoglycan form of dentin matrix protein 1 (DMP1-PG) leads to the downregulation of the JAK2/STAT3 signaling pathway, which affects the differentiation of MSCs into chondrocytes [58]. In this study, the JAK 1/2/3 transcription level of h-JBMMSCs changed significantly after knocking down or overexpressing the lncRNA SNHG1, and only STAT3 in STATs changed significantly with the expression of the lncRNA SNHG1, therefore, STAT3 was selected for further study. iJAK is an inhibitor of JAK family kinases. Compared with that in untreated cells, the level of the downstream target of JAK, iJAK, significantly reduced the level of phosphorylated STAT3. Moreover, to verify the regulatory effect of JAK on chondrogenic differentiation, the use of iJAK significantly inhibited Alcian blue staining during cartilage-induced differentiation. These results suggest that in h-JBMMSCs, the lncRNA SNHG1 promotes STAT3 phosphorylation through JAK, thus promoting chondrogenic differentiation. STATs function as effectors of mitochondrial function, and STAT3 is a member of seven protein families that play key roles in the signal transduction of cytokines and growth factors [57]. STAT3 plays an important role outside the nucleus, including in mitochondria. STAT3 reportedly supports RAS-dependent carcinogenesis and participates in cell respiration [59]. Nine of the 14 cysteine residues in STAT3 are sensitive to redox reactions and can be directly modified by oxidative stress. Szczepanek et al. [60] showed that mitochondrial STAT3 can prevent stress-induced changes in the electron transfer chain, leading to the generation of ROS. Serine-phosphorylated STAT3 (pS-STAT3) interacts with electron transfer chain (ETC) complexes I and ETCII, increases membrane polarization and ATP production, and enhances the activity of lactate dehydrogenase, thus increasing aerobic glycolysis and reducing ROS production [61]. Therefore, it is speculated that the lncRNA SNHG1 may regulate mitochondrial function through STAT3 phosphorylation in h-JBMMSCs.

Mitochondria participate in the regulation of MSCs differentiation through energy metabolism, antioxidant pathways, mitochondrial biogenesis, and mitochondrial dynamics [62, 63]. Therefore, we detected cROS by DHE staining and intracellular mROS levels by MitoSOX staining. The probe itself has no fluorescence, and after entering the cell, it is oxidized by ROS to generate fluorescent substances. When the lncRNA SNHG1 is overexpressed, the fluorescence intensity is reduced and the ROS level is reduced. Moreover, mitochondrial membrane potential and ATP content detection showed that the lncRNA SNHG1 increased the MMP and ATP production in JBMMSCs. A normal MMP is an important condition for maintaining the oxidative phosphorylation of mitochondria to produce ATP [64]. The proton pump in the inner membrane of mitochondria pumps protons (H^+^) in the matrix into the membrane gap, forming a transmembrane potential across the inner membrane of mitochondria, that is, the MMP [62, 65]. When protons return, ATP is produced. The greater the MMP is, the greater the ATP production rate [66, 67]. When the ROS concentration is too high, it will destroy the mitochondrial membrane, change the permeability of the membrane, reduce the concentration difference of ions inside and outside the membrane, and lead to a decrease in the membrane potential and ATP synthesis [62, 68]. In addition, ROS can also attack mitochondrial DNA to produce oxidative damage [65].

Mitochondrial biogenesis is characterized by an increase in mitochondrial DNA (mtDNA) copy number and mitochondrial gene expression. This process is coordinated by the transcription coactivator PGC-1, and PGC-1α is the main regulator that regulates mitochondrial biogenesis by binding to the nuclear receptor in the promoter region of the target gene and specific sequences [69]. PGC-1α regulates the transcription of TFAM after coactivating Nrf1 and 2 [70]. TFAM translocates to the mitochondrial matrix and stimulates mtDNA replication and mitochondrial gene expression [71]. Therefore, the mRNA levels of PGC-1α, Nrf1, and TFAM in h-JBMMSCs after knocking down or overexpressing the lncRNA SNHG1 were detected. The results showed that the lncRNA SNHG1 promoted the transcription of various factors related to mitochondrial biogenesis, suggesting that the lncRNA SNHG1 might promote the mitochondrial biogenesis in h-JBMMSCs. Although these results show that the lncRNA SNHG1 regulates mitochondrial function, the effect of mitochondrial function on the chondrogenic differentiation of h-JBMMSCs is still unclear. Therefore, we added the ROS inhibitor NAC during chondrogenic differentiation, which significantly alleviated the inhibition of chondrogenic differentiation caused by sh-SNHG1. The lncRNA SNHG1 can affect the chondrogenic differentiation of h-JBMMSCs by regulating ROS levels.

## Conclusions

The lncRNA SNHG1 can promote chondrogenic differentiation and angiogenesis of h-JBMMSCs. The lncRNA SNHG1 regulates the mitochondrial function of h-JBMMSCs by reducing ROS levels, enhancing mitochondrial membrane potential, increasing ATP levels, and promoting cartilage regeneration. On the other hand, the lncRNA SNHG1 enhances the phosphorylation of the STAT3 protein through JAK, thus promoting chondrogenic differentiation. Combined with the studies on the regulation of mitochondria by STAT3 phosphorylation, it is speculated that the lncRNA SNHG1 could reduce ROS production by increasing STAT3 phosphorylation, thus promoting cartilage regeneration in h-JBMMSCs, which will be further studied in the future. These findings provide a new target for promoting cartilage differentiation of h-JBMMSC, and have potential application value in cartilage regeneration and repair. These findings provide a new target for promoting h-JBMMSC chondrogenic differentiation, and targeting lncRNA SNHG1 or its associated signaling pathways may be a viable approach for cartilage regeneration in patients with post-traumatic cartilage defects. Based on the current therapeutic lncRNA targeting methods, including antisense oligonucleotides oligonucleotide, small molecule targeting lncRNA and CRISPR-Cas tool. CRISPR-Cas is the most likely tool to be applied and accurately regulated in the future for this study, CRISPRa could be used to activate the coding gene of lncRNA SNHG1 in JBMMSC for cartilage regeneration. Or consider how to induce JBMMSC to differentiate into cartilage tissue in vitro and use it for transplantation in vivo. In addition, the regulation of lncRNA SNHG1 on mitochondria also suggests that the differentiation and application of stem cells can be regulated from organelles. In addition, there are still some limitations in this study, such as the regulatory effect of lncRNA SNHG1 on blood vessels through JAK/STAT, which needs further study, and the study of TMJ cartilage defect model will be more comprehensive.

### Supplementary Information

Additional file 1.

Additional file 2.

## Data Availability

All the data generated or analyzed during this study are included in this published article. The datasets used and analyzed in the current study are available from the corresponding author upon reasonable request.

## Abbreviations

Ang: Angiopoietin
ATP: Adenosine triphosphate
BMSCs: Bone marrow mesenchymal stem cells
CDH5: VE-cadherin
CDKN1B: Cyclin dependent kinase inhibitor 1B
COL2: Collagen II
COL5: Collagen V
CS: Chondroitin sulfate
DHE: Dihydroethidium
DMP1-PG: Dentin matrix protein 1
EGFR: Epidermal growth factor receptor
ETC: Electron transfer chain
FGF-2: Fibroblast growth factor-basic
GAG: Glycosaminoglycan
GuHCl: Guanidine hydrochloride
h-JBMMSCs: Human jaw bone marrow mesenchymal stem cells
HUVECs: Human umbilical vein endothelial cells
ICRS: International Cartilage Repair Society
IF: Immunofluorescence
IFNGR1: Interferon gamma receptor 1
IHC: Immunohistochemistry
JAK: Janus kinase
KLF2: Kruppel-like factor 2
LncRNAs: Long non-coding RNAs
Mettl3: Methyltransferase like 3
MFI: Mean fluorescence intensity
MSCs: Mesenchymal stem cells
MMP, △ψm: Mitochondrial membrane potential
mtDNA: Mitochondrial DNA
NAC: N-acetylcysteine
Nrf: Nuclear respiratory factor
PBS: Phosphate-buffered saline
PGC-1: Peroxisome proliferator-activated receptor 1
PGs: Glycoproteins
PMSF: Phenylmethanesulfonyl fluoride
pS-STAT3: Serine-phosphorylated STAT3
PTBP1: Polypyrimidine tract binding protein 1
PVDF: Polyvinylidene fluoride
ROS: Reactive oxygen species
SIRT1: Silent information regulator 1
SMSCs: Synovium-derived mesenchymal stem cells
STAT: Signal transducers and activators of transcription
TFAM: Mitochondrial transcription factor A
VEGF: Vascular endothelial growth factor
