# Supplementary material for: LncRNA SNHG1 enhances cartilage regeneration by modulating chondrogenic differentiation and angiogenesis potentials of JBMMSCs via mitochondrial function regulation
Source: Stem Cell Res Ther. 2024 Jun 18;15:177. doi: 10.1186/s13287-024-03793-2 (PMC11184886; doi:10.1186/s13287-024-03793-2)
Supplement: Supplementary file 1 — Additional file 1. [file 13287_2024_3793_MOESM1_ESM.docx]

Supplementary Table 1. Primers sequences used in the real-time RT-PCR

| Gene Symbol | Primer Sequences (5’-3’) |
| --- | --- |
| LncRNA SNHG1 | Forward: ACAGCAGTTGAGGGTTTGCT |
|  | Reverse: GGGCCTGGATCATGTAAGAA |
| COL2 | Forward: TGGACGATCAGGCGAAACC |
|  | Reverse: GCTGCGGATGCTCTCAATCT |
| COL5 | Forward: GACTGTGCCGACCCTGTAAC |
|  | Reverse: CCTGGACGACCACGTATGC |
| SOX9 | Forward: AGCGAACGCACATCAAGAC |
|  | Reverse: CTGTAGGCGATCTGTTGGGG |
| VEGF | Forward: AGGGCAGAATCATCACGAAGT |
|  | Reverse: AGGGTCTCGATTGGATGGCA |
| Ang | Forward: ACCTCACCCTGCAAAGACAT |
|  | Reverse: TCCATGTAGCTTGCAAGTGG |
| CD31 | Forward: AAGCTGCCGGTTCTTAAATCC |
|  | Reverse: AACTTGGTGGAAGGAGGGTATG |
| CDH5 | Forward: AAGCGTGAGTCGCAAGAATG |
|  | Reverse: TCTCCAGGTTTTCGCCAGTG |
| FGF2 | Forward: AGAAGAGCGACCCTCACATCA |
|  | Reverse: CGGTTAGCACACACTCCTTTG |
| PGC-1α | Forward: TCTGAGTCTGTATGGAGTGACAT |
|  | Reverse: CCAAGTCGTTCACATCTAGTTCA |
| Nrf1 | Forward: AGGAACACGGAGTGACCCAA |
|  | Reverse: TATGCTCGGTGTAAGTAGCCA |
| TFAM | Forward: ATGGCGTTTCTCCGAAGCAT |
|  | Reverse: TCCGCCCTATAAGCATCTTGA |
| JAK1 | Forward: TGCAACACTTGAAAGACCCG |
|  | Reverse: TTGCTGCCACTGAACCAATC |
| JAK2 | Forward: TCTGGGGAGTATGTTGCAGAA |
|  | Reverse: AGACATGGTTGGGTGGATACC |
| JAK3 | Forward: CTGTTGACTGGCTGTGTGAC |
|  | Reverse: TGCCCATCTGTCTTGAACCT |
| STAT1 | Forward: GTATGCCATCCTCGAGAGCT |
|  | Reverse: TACCACTGAGACATCCTGCC |
| STAT2 | Forward: TGATGGACCCTTGATGCCTT |
|  | Reverse: TTGTTGTCCCCTCTGTACCC |
| STAT3 | Forward: CGGAAGAGAGTGCAGGATCT |
|  | Reverse: CAGCTCACTCACGATGCTTC |
| GAPDH | Forward: CCAGGTGGTCTCCTCTGA |
|  | Reverse: GCTGTAGCCAAATCGTTGT |
