# Supplementary material for: LncRNA SNHG1 enhances cartilage regeneration by modulating chondrogenic differentiation and angiogenesis potentials of JBMMSCs via mitochondrial function regulation
Source: Stem Cell Res Ther. 2024 Jun 18;15:177. doi: 10.1186/s13287-024-03793-2 (PMC11184886; doi:10.1186/s13287-024-03793-2)
Supplement: Supplementary file 2 — Additional file 2. [file 13287_2024_3793_MOESM2_ESM.docx]

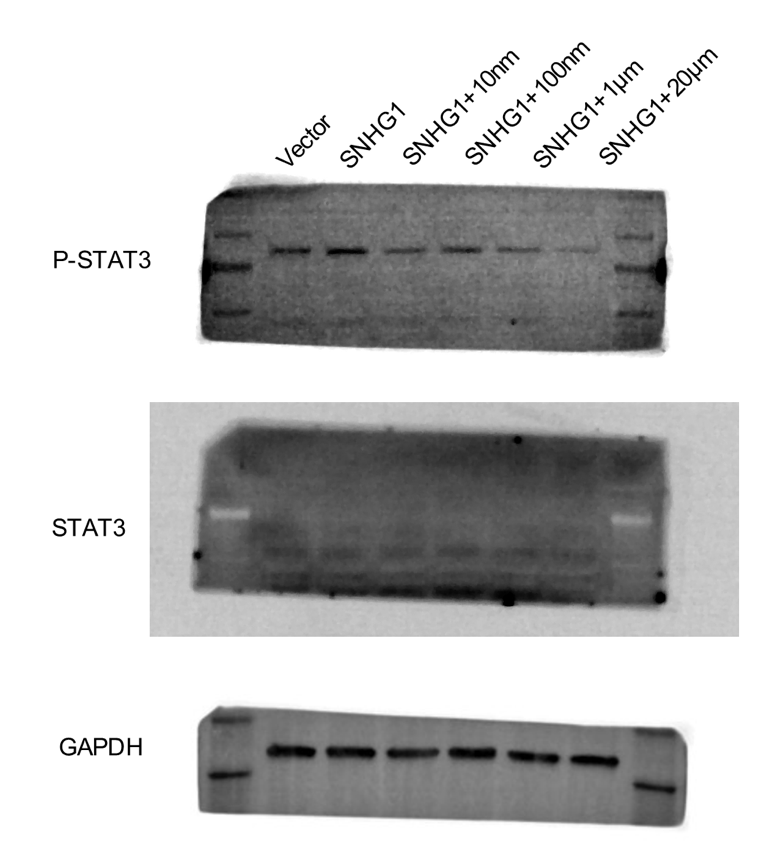


**Additional file 2:** **Original western blot gels**

Original western blot gels of Fig. 8M for protein expression levels of P-STAT3 and STAT3 in different groups.
